# Antibacterial and antioxidant okra-based hydrogels enhanced with Ag@HKUST-1 as a next-generation wound dressing

**DOI:** 10.1016/j.ijpx.2026.100633

**Published:** 2026-08-20

**Authors:** Shabnam Tahmasebi, Hassan Farmanbordar, Reza Mohammadi

**Affiliations:** Polymer Research Laboratory, Department of Organic and Biochemistry, Faculty of Chemistry, University of Tabriz, Tabriz, Iran

**Keywords:** Wound dressing, Okra polysaccharide, Silver nanoparticle, Antibacterial, Superabsorbent

## Abstract

Wound management represents a significant global challenge. This study aims to develop an innovative multifunctional Okra/PAAm/PAA/Ag@HKUST-1 hydrogel to enhance wound dressing properties. The hydrogel was synthesized by functionalizing okra polysaccharide with dialdehyde/dicarboxylate groups, followed by polymerization of acrylamide and acrylic acid. This method yielded a super absorbent material with notable swelling properties, effectively controlling wound exudates while maintaining a moist healing environment. The hydrogel gained impressive antibacterial and antioxidant properties by integrating HKUST-1 and silver nanoparticles (Ag NPs), which Ag NPs were synthesized and stabilized by *Calendula officinalis* (CO) extract. Characterization by FT-IR, SEM, and XRD verified that the hydrogel was successfully synthesized. The Hydrogel/Ag@HKUST-1 composite demonstrated notable bacteria-inhibiting activity toward *E. coli (*inhibition zones ∼18 mm*)* and *S. aureus* (inhibition zone ∼22 mm) and strong antioxidant capacity, with an activity level of 52.06%. Hemolytic activity and cytotoxicity assays on HFF-2 cells showed minimal hemolysis (0.58%) and low cytotoxicity (99.21% at 24 h and 98.54% at 48 h). The hydrogel's blood clotting test demonstrated pro-coagulant characteristics (BCI = 22%) for quick bleeding stoppage and improved wound closure. These findings emphasize the Hydrogel/Ag@HKUST-1's promise as a potent wound dressing.

## Graphical abstract

**Figure:**
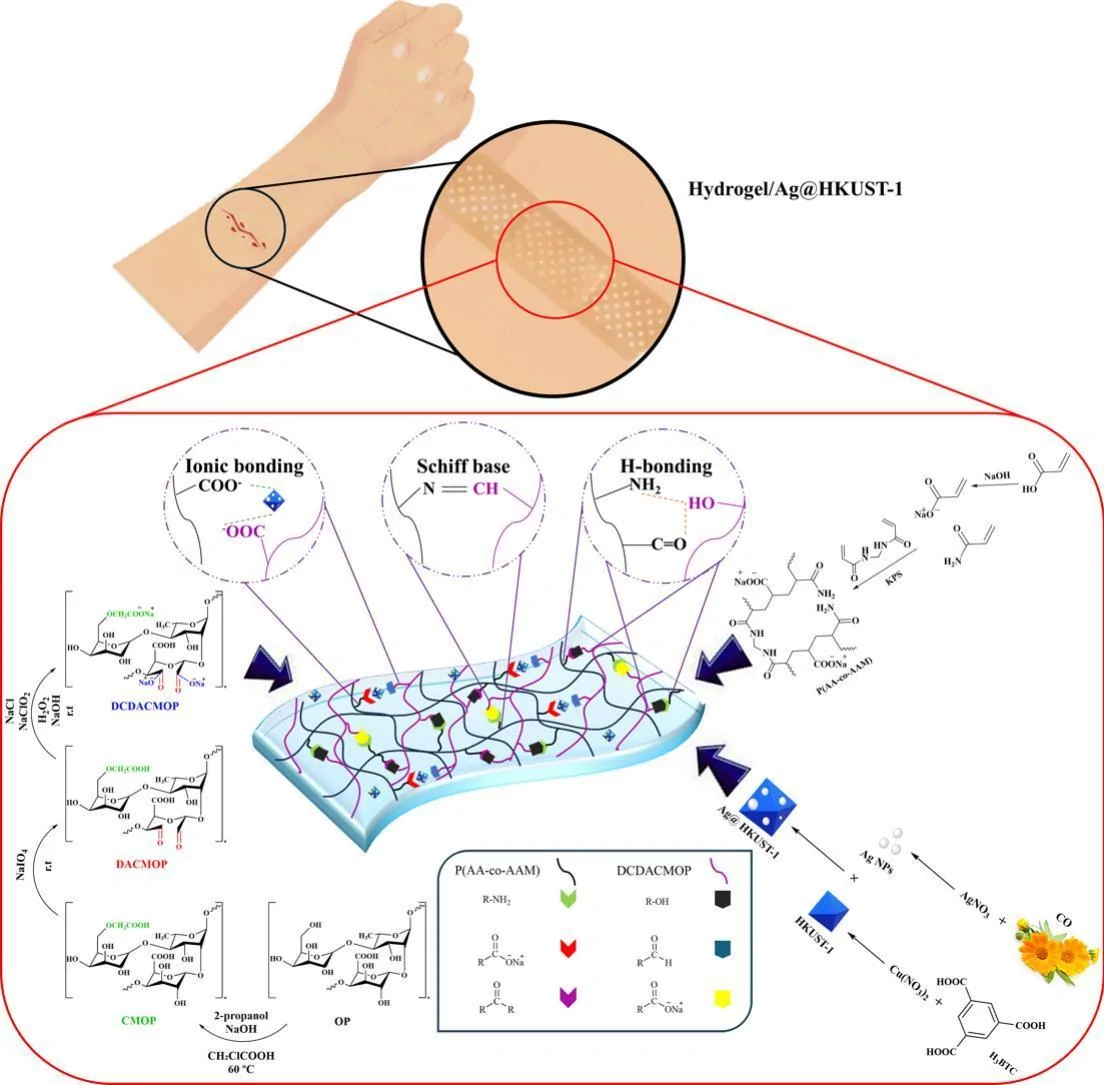

## 1 Introduction

Wound management constitutes a considerable global challenge stemming from the associated risks of infection, the treatment's elevated financial burden, and tissue injury linked with conventional methods like sutures and staples (Byrne and Aly, 2019). As a result, innovative, multifunctional wound dressings have achieved significant interest in addressing these limitations, particularly those made from hydrogels composed of polysaccharides. This interest is attributed to their biocompatibility, ability to mimic the extracellular matrix (ECM), capacity to retain biological fluids, facilitation of therapeutic agent delivery, and potential for modification to include antioxidant and antibacterial properties (Cai et al., 2023; Lei et al., 2021; Ouyang et al., 2023; Ullah et al., 2025).

Natural polysaccharides, such as those found in okra (*Abelmoschus esculentus* L.), are noted for their potential in wound healing applications due to their rich content of bioactive compounds, including antioxidants and flavonoids. These compounds are key in controlling oxidative stress, as they neutralize damaging reactive oxygen species (ROS) and encourage the release of cytokines and growth factors involved in tissue regeneration (Ilmi et al., 2020; Liang et al., 2025; Liao et al., 2012). However, the lack of mechanical strength is a limitation of polysaccharide-based hydrogels that restricts their broader application (Bao et al., 2019). To tackle this challenge, polysaccharide-based hydrogels are chemically modified with a wide variety of functional group and various polymers and polysaccharides to enhance mechanical stability, dispersibility and introduce properties such as self-healing (Yang and Wang, 2023). Carboxymethylation of polysaccharide improves swelling index and re-dispersibility (Alalor and Obunezie, 2020). Moreover, dialdehyde of polysaccharide enables a wide choice of cross-linking agents (Yi et al., 2022). Utilizing polymers such as polyacrylamide (PAAm) and polyacrylic acid (PAA) for chemical modification enhances not only the mechanical stability of hydrogels but also their moisture retention, antibacterial properties, and ability to promote collagen synthesis, thereby accelerating tissue regeneration (Ferreira et al., 2023; Shahrousvand et al., 2023).

Additionally, the frequent use of antibiotics in wound care has led to some bacteria developing resistance (Sun et al., 2022). Therefore, integrating green-synthesized nanoparticles (NPs), such as silver nanoparticles (Ag NPs), TiO₂, and ZnO, into hydrogel structures offers a promising way to combat bacterial resistance due to their antibacterial properties (Bai and Jarubula, 2023; Cai et al., 2025; Nandhini et al., 2023; Sivaranjani and Philominathan, 2016). Moreover, plant extracts such as *Calendula officinalis* extract (COE) and cinnamon, which are used to stabilize and green synthesis NPs, can prevent NPs agglomeration during synthesis and improve their stability and effectiveness (Alanazi, 2024; Alipour et al., 2025; Ghasemi et al., 2022). About Ag NPs, they exert their primary antibacterial effect through a multi-stage mechanism: they first compromise the structural integrity of the bacterial cell wall, penetrate the plasma membrane, and subsequently disrupt key metabolic processes. This potent bactericidal action, coupled with their ability to modulate inflammation, makes them a highly effective agent in promoting wound healing (Fox, 1968; Hoffmann, 1984; Lansdown, 2010; Lara et al., 2010; Nadworny et al., 2008). HKUST-1, a porous metal-organic framework (MOF) containing copper (Cu) ions, has demonstrated antibacterial, antifungal, and wound healing properties (Chaturvedi and Henderson, 2014; Davoodian et al., 2025). By stimulating the synthesis of vascular endothelial growth factor (VEGF), these MOFs actively promote angiogenesis - the growth of new blood vessels - which is a critical step in facilitating faster and more effective tissue repair (Jacobs et al., 2020). Moreover, recent MOF-based composite hydrogels have shown promising antibacterial activity and accelerated wound healing through controlled release and microenvironment regulation (Guo et al., 2025).

**Scheme 1:**
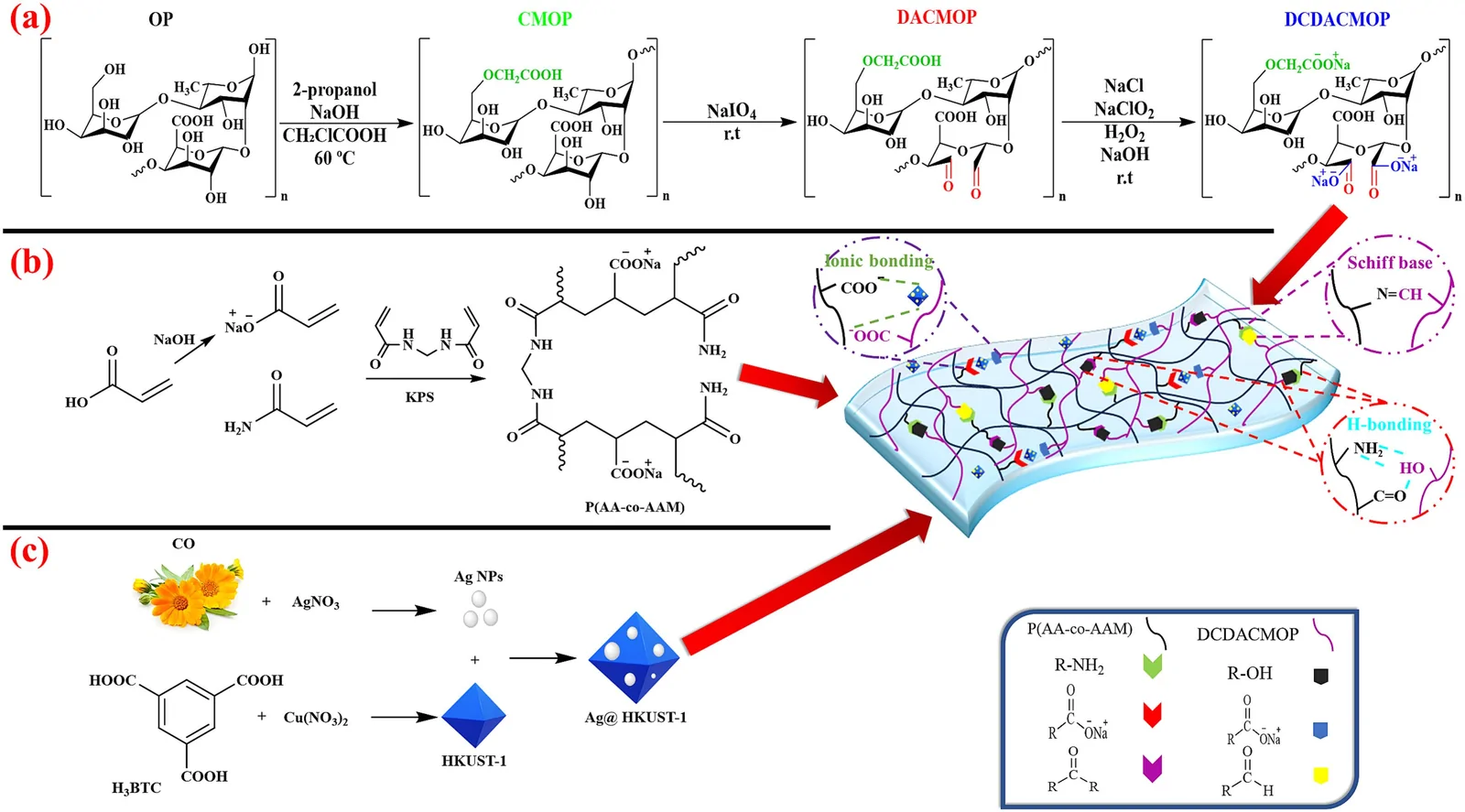
A schematic overview of the integrated synthesis for producing adhesive, superabsorbent, antioxidant, and antibacterial hydrogel composites. (a) details the chemical modification pathway to obtain DCDACMOP. (b) shows the preparation of the poly (AA-*co*-AAm) hydrogel network. (c) summarizes the procedure for synthesizing Ag@HKUST-1 nanoparticles.

This study aims to develop novel multifunctional Okra/PAAm/PAA/Ag@HKUST-1 hydrogel (Hydrogel/Ag@HKUST-1) scaffolds for wound dressing applications (Scheme 1). This innovative system was formed through dynamic Schiff base bonds and hydrogen bonds within a precisely designed hydrogel network. Ag NPs, serving as both antibacterial and antioxidant agents, are green-synthesized and stabilized by COE extract. The incorporation of HKUST-1 enhances antibacterial and antioxidant properties in a hydrogel matrix, thereby increasing cell compatibility and accelerating blood clotting index. Additionally, various combinations of Ag@HKUST-1 were evaluated to examine their effect on the resulting hydrogel's physicochemical properties and its functional biological response.

## 2 Experimental section

### 2.1 Materials

Fresh okra (*Abelmoschus esculentus*) and calendula (*Calendula officinalis*) were sourced from a local market in Tabriz, Iran. All chemical reagents, including acrylamide (AAm), silver nitrate (AgNO₃), potassium persulfate (KPS), and acrylic acid (AA), were acquired from Sigma-Aldrich. A comprehensive list of chemicals comprises: chloroacetic acid, sodium chlorite, sodium periodate, *N*, *N*′-methylenebisacrylamide (MBA), dimethylformamide (DMF), hydrochloric acid (HCl), hydrogen peroxide (H₂O₂), copper (II) nitrate pentahydrate (Cu (NO₃)₂·5H₂O), trimesic acid, ethanol (97%), ethylene glycol, 2-propanol, and DPPH (2,2-diphenyl-1-picrylhydrazyl).

### 2.2 Methods used to characterize materials

The compositional and structural properties of the synthesized materials—calendula/okra (CO) extract, silver nanoparticles (Ag NPs), HKUST-1, the composite Ag@HKUST-1, and the final hydrogel were analyzed using a suite of characterization techniques. These included Fourier transform infrared spectroscopy (FTIR), energy-dispersive X-ray spectroscopy (EDX), X-ray diffraction (XRD), and scanning electron microscopy (SEM). Full details on the instrumental parameters and analytical procedures are presented in the Supplementary Information (SI).

### 2.3 Synthesis of carboxymethyl okra polysaccharide (CMOP)

Okra polysaccharide (OP) was initially extracted from fenugreek seeds according to a published method (see SI) (Rashid et al., 2018). Subsequently, carboxymethylation was performed as follows: 2 g of OP were suspended in 2-propanol (100 mL) and stirred at an ambient temperature for an hour. A separate aqueous solution containing 4 g of NaOH in distilled water (100 mL) was then added to the mixture, with stirring maintained at 40 °C for an additional hour. Following this, 0.5 g (5.29 mmol) of chloroacetic acid was added, and the reaction proceeded at 60 °C for 2 h to facilitate etherification. The resultant CMOP was isolated by extensive washing with ethanol to remove by-products and salts, followed by drying. The carboxymethylation efficiency, determined by acid-base titration (see SI), was approximately 78% (Behrouzi and Moghadam, 2018; Huang et al., 2020; Lin et al., 2021; Stojanović et al., 2005).

### 2.4 Preparation of dialdehyde CMOP (DACMOP)

Dialdehyde CMOP was prepared by oxidizing the hydroxyl functionalities of the polysaccharide with sodium periodate (NaIO₄) (Lucas de Lima et al., 2020). Briefly, a solution of CMOP (1 g in 50 mL H₂O) was reacted with NaIO₄ (0.5 g, 2.33 mmol) overnight at room temperature under light-protected conditions (aluminum foil wrap). The reaction was quenched with a stoichiometric equivalent of ethylene glycol, stirred for 6 h, and the product was isolated via ethanol washing, centrifugation, and drying at 50 °C. Titration of the formed aldehyde groups using hydroxylamine hydrochloride confirmed an oxidation efficiency of ∼80% (see SI for method) (Lucas de Lima et al., 2020; Sichinga et al., 2022).

### 2.5 Preparation of di-carboxylate DACMOP (DCDACMOP)

Di-carboxylate DACMOP (DCDACMOP) was synthesized via the oxidation of DACMOP's aldehyde groups using a sodium chlorite (NaClO₂) system. A solution of DACMOP (1 g in 60 mL H₂O) was treated sequentially with NaCl (2.93 g), NaClO₂ (0.3 g), and H₂O₂ (0.02 g). The pH was buffered to 5 with 0.5 M NaOH, and the reaction proceeded overnight under ambient stirring. This oxidation step yielded a conversion efficiency of roughly 79%, quantified by titration methods (refer to SI) (Yang et al., 2013).

### 2.6 Biosynthesis of Ag NPs

Silver nanoparticles were synthesized using a green chemistry approach, employing a calendula/okra (CO) extract as both the reductant and capping agent. The extract, prepared by maceration (SI), was reacted with an aqueous silver nitrate solution (1 g extract, 0.5 g AgNO₃ in 20 mL H₂O) at 65 °C for 3 h (A color shift to light brown indicates the reaction progress). The Ag NPs were subsequently centrifuged, washed with d-water, and oven-dried (50 °C).

### 2.7 Preparation of Ag@HKUST-1 composite

To prepare the Ag@HKUST-1 composite, pre-synthesized HKUST-1 (See SI) (Nivetha et al., 2020) and biosynthesized Ag NPs were combined in a 4:1 ratio in DMF solvent. After 30 min of stirring for initial dispersion, the reaction proceeded for 3 h at 70 °C. The final composite was obtained by filtration, washed with DMF, and dried at ambient temperature (Asadevi et al., 2022).

### 2.8 Preparation of Hydrogel/Ag@HKUST-1

The Hydrogel/Ag@HKUST-1 composite was prepared through copolymerization of acrylic acid (AA) and acrylamide (AAm). First, AA (1.29 g, 18 mmol) was neutralized in a cold NaOH solution. Acrylamide (1.27 g, 18 mmol) and the cross-linker MBA (0.03 g) were then added. Concurrently, a solution of DCDACMGM (1 g) and Ag@HKUST-1 nanoparticles was prepared and combined with the monomer mixture. Following 15 min of deoxygenation under N₂, polymerization was initiated with potassium persulfate (KPS, 0.01 g). The formed hydrogel composite was finally washed with ethanol and vacuum-dried (see Scheme 1).

### 2.9 Lab-based swelling and disintegration study

To simulate performance in a biological environment, the hydrogels' swelling behavior was investigated. The dried samples were incubated in phosphate-buffered saline (PBS, pH 7.4) at 37 °C to mimic physiological conditions (Lv et al., 2018; Nezhad-Mokhtari et al., 2024). The resulting swelling ratio (SR%), calculated via Eq. (1), served as the key metric for absorption capacity.

(1) $$\text{Swelling ratio}\;\left(\mathrm{SR}\%\right)=\frac{w_{2-}w_{1}}{w_{1}}\times 100$$

The samples' initial dry and swollen weights are W_1_ and W_2_, respectively.

Degradation studies were conducted by incubating pre-weighed dry hydrogel samples in PBS (pH 7.4) at 37 °C under constant shaking (100 rpm). At predetermined time intervals, the samples were retrieved, washed with distilled water (dH₂O), and dried to achieve a constant weight. The degradation rate was calculated according to Eq. (2):

(2) $$\text{Weight remaining ratio}\;\left(\%\right)=\frac{w_{t}}{w_{0}}\times 100$$

In which W_0_ is the dry primary weight and W_t_ is the dry post-degradation weight at time “t”.

### 2.10 Assessment of adhesive properties

The hydrogels adhesive capability was investigated across a variety of common surfaces to simulate potential application environments. Samples were qualitatively tested on substrates including glass, steel, plastic, human skin, and wood under ambient conditions, with the effectiveness of their interfacial bonding documented.

### 2.11 Free radical–scavenging activity

The free radical–scavenging activity of the materials was assessed through the 2,2-diphenyl-1-picrylhydrazyl (DPPH) radical scavenging assay (Gardeli et al., 2008). First, each hydrogel sample (0.01 g) was rehydrated in distilled water (1 mL) and gently agitated for 12 h to encourage the release of active constituents. Following this, 0.01 g aliquots of the CO extract, HKUST-1, Ag NPs, Ag@HKUST-1, and the hydrogel eluent were individually combined with 2 mL of a 0.2 mM DPPH solution in ethanol. All resultant combinations were incubated at ambient temperature and under light-protected conditions for 30 min to reduce the photodegradation of the DPPH radicals. UV–Vis spectrophotometer was utilized to record the absorbance (at 517 nm), and the radical scavenging activity (RSA %) was calculated using Eq. (3):

(3) $$\mathrm{DPPH}\;\mathrm{RSA}\;\left(\%\right)=\frac{A_{B}-A_{A}}{A_{B}}\times 100$$

In the eq. (3), *A*_*B*_ signifies the blank solution absorbance of the DPPH and *A*_*A*_ denotes the sample-containing solution absorbance.

### 2.12 Evaluation of antibacterial activity

#### 2.12.1 Determination of MIC and MBC

The antibacterial effectiveness of the CO extract, Ag NPs, HKUST-1, and the Ag@HKUST-1 composite were examined against *S. aureus* and *E. coli*. Minimum inhibitory concentrations (MICs) were identified using a twofold serial dilution approach in Mueller–Hinton broth within a 96-well microplate format. Each well received 10 μL of a bacterial inoculum adjusted to 1.5 × 10^8^ CFU/mL, followed by the addition of 20 μL of a 0.1% resazurin solution, which served as an indicator of cellular metabolic activity. Plates were incubated at 37 °C for 24 h, after which the MIC was recorded as the lowest sample concentration that produced no visible color transition. To determine the minimum bactericidal concentration (MBC), aliquots from wells showing inhibited growth were streaked onto BHI agar and incubated at 37 °C for an additional 24 h.

#### 2.12.2 Agar well diffusion assay

The agar well diffusion technique against *E. coli* and *S. aureus* was conducted to evaluate antimicrobial performance of CO, HKUST-1, Ag NPs, Ag@HKUST-1, the hydrogel, and the Hydrogel/Ag@HKUST-1 composite (Nia et al., 2020). Mueller–Hinton agar plates were uniformly seeded with bacterial suspensions (1.5 × 10^8^ CFU/mL) using sterile cotton swabs. A sterile borer used to punch wells aseptically into the agar, and each was carefully filled with the respective hydrogel formulations. Then, the plates at 37 °C were incubated for 24 h. At the end of the incubation period, the diameters of the inhibition zones were recorded with a digital caliper to quantify the antibacterial activity.

### 2.13 Hemolysis in vitro assay

To ensure the hydrogels are safe for contact with blood, their hemocompatibility was tested by measuring red blood cell rupture (hemolysis) (Nezhad-Mokhtari et al., 2024). For this assay, 5 mg/mL hydrogel samples at 37 °C were pre-incubated in PBS (pH 7.4) for 20 min. An equal volume of each sample solution was then mixed with diluted human blood and incubated at 37 °C for one hour. Following incubation, the mixtures were centrifuged (800 rpm) for 4 min (The supernatants absorbance was measured at 540 nm). Deionized water and PBS alone served as positive (complete hemolysis) and negative (no hemolysis) controls, respectively. The hemolysis percentage was evaluated via Eq. (4):

(4) $$\text{Hemolsis}\;\left(\%\right)=\frac{A_{s-}A_{p}}{A_{w-}A_{p}}\times 100$$

Since A_S_ is the sample absorbance, and A_W_, A_P_ are the positive and negative control absorbance, respectively.

### 2.14 Blood clotting

The coagulation activity of the hydrogels was investigated to determine their potential in managing bleeding (Guo et al., 2022; Shakya et al., 2019). The blood clotting index (BCI) was determined as follows: Fresh human blood from a consenting donor was collected in an acid citrate dextrose (ACD) anticoagulant solution. Pre-weighed hydrogel samples (25 mg) were placed in warm (37 °C) petri dishes and coated uniformly with 0.25 mL of the anticoagulated blood. Coagulation was initiated after 15 min by adding 25 μL of 0.2 M calcium chloride solution. At set time points (3, 5, 10, 15, 20, and 30 min), 10 mL of d-water was gently increased to each dish to lyse any free red blood cells not trapped in the clot. The supernatant was collected by centrifuging of the solutions, diluted, and at 37 °C incubated for 70 min (absorbance was measured at 542 nm). A reference sample of ACD-treated blood diluted in water was assigned an absorbance value of 100%. The BCI was calculated using Eq. (5):

(5) $$\mathrm{BCI}=\frac{A_{t}}{A_{0}}\times 100$$

where lower values indicate superior clotting performance due to reduced free hemoglobin (A_t_) compared to a reference standard (A_0_).

### 2.15 MTT assay for cytotoxicity evaluation

The potential toxicity of the hydrogels was evaluated on human fibroblast cells (HFF-2) using the MTT colorimetric assay, a standard measure of metabolic activity (Boukani et al., 2022). After UV sterilization, hydrogel samples were placed in contact with pre-cultured cells for 24 and 48 h. Living cells metabolize the yellow MTT reagent, producing purple formazan crystals. Following treatment, these crystals were dissolved in DMSO, and the intensity of the resulting purple color, measured at 570 nm, is directly proportional to the number of viable cells. This allows for a quantitative comparison of cell health between hydrogel-treated samples and untreated (negative) or toxic (positive) controls. All quantitative data are presented as mean ± standard deviation (SD). Statistical analyses were performed using one-way analysis of variance (ANOVA), followed by an appropriate post hoc multiple-comparison test. Differences were considered statistically significant at *p* < 0.05.

## 3 Results and discussion

### 3.1 FTIR and XRD studies

Fourier-transform infrared (FTIR) spectroscopy, UV–Vis spectroscopy, and X-ray diffraction (XRD) were employed to confirm the chemical structures and phases of the synthesized materials. The analyses focused on two primary tracks: (i) the formation of the Ag@HKUST-1 nanocomposite, and (ii) the chemical modification of okra polysaccharide (OP) and its subsequent incorporation into the final hydrogel.

**Fig. 1:**
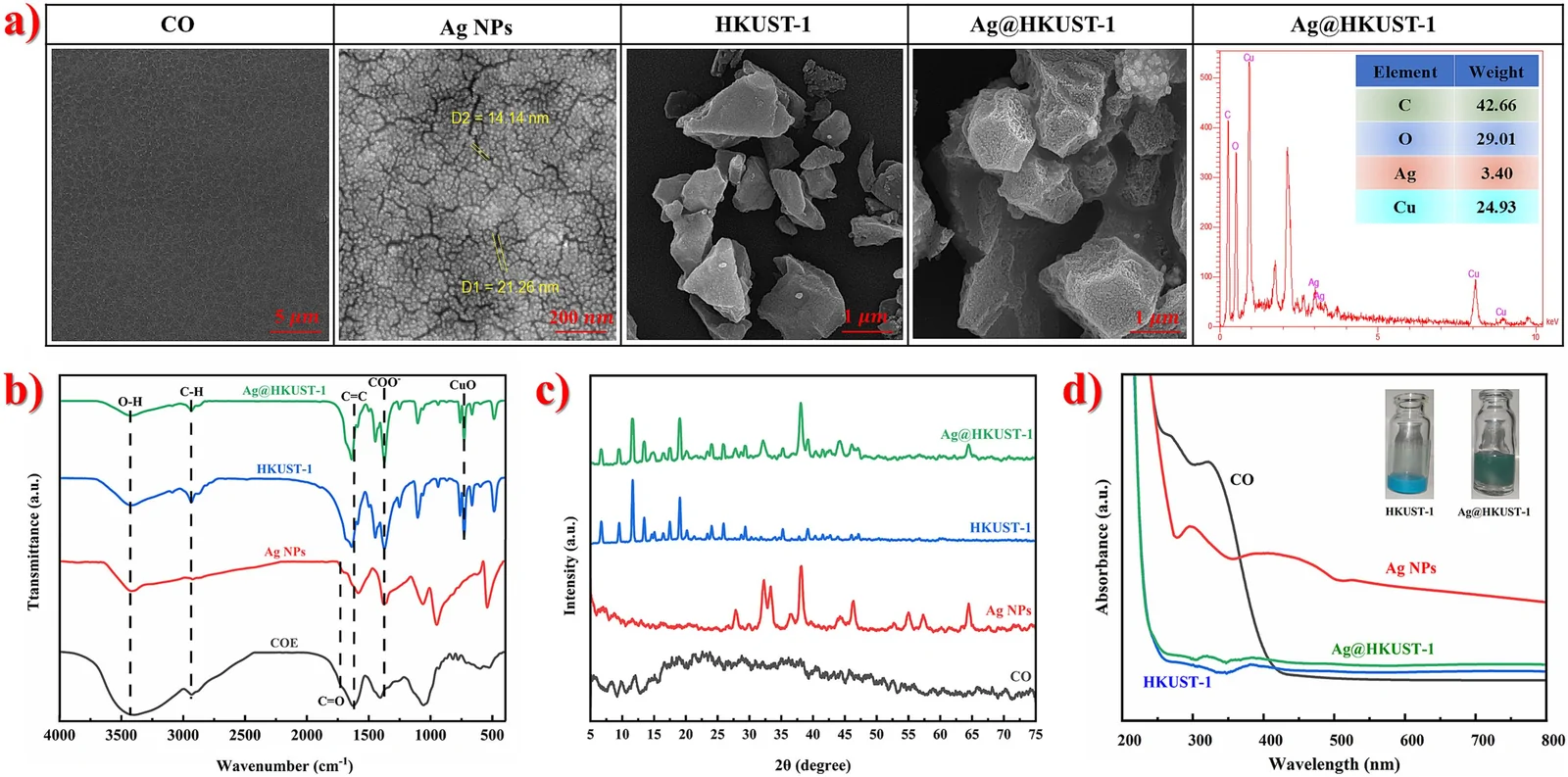
a) SEM images of CO, Ag NPs, HKUST-1, Ag@HKUST-1, and EDX image of Ag@HKUST-1. b) FTIR spectra for CO extract, AgNPs, HKUST-1, Ag@HKUST-1. c) XRD patterns for CO extract, AgNPs, HKUST-1, Ag@HKUST-1. d) UV–Vis absorption spectra of CO extract, AgNPs, HKUST-1, Ag@HKUST-1.

**Fig. 2:**
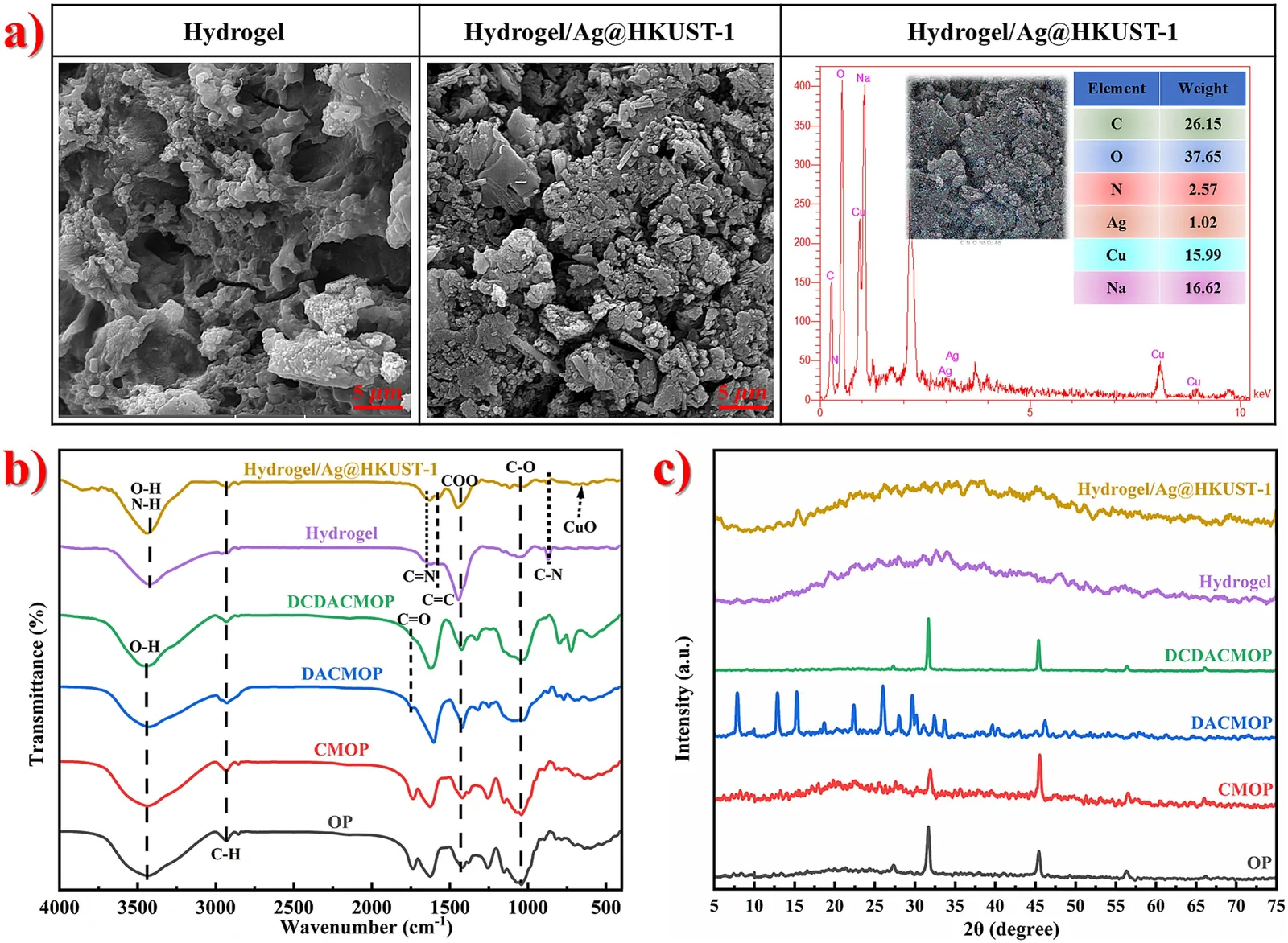
a) SEM image of Hydrogel, Hydrogel/Ag@HKUST-1, and EDX/MAP image of Hydrogel/Ag@HKUST-1. b) FTIR spectra of OP, CMOP, DACMOP, DCDACMOP, Hydrogel, and Hydrogel/Ag@HKUST-1. c) XRD patterns of OP, CMOP, DACMOP, DCDACMOP, Hydrogel, and Hydrogel/Ag@HKUST-1.

The FTIR spectra for all materials are presented in Fig. 1b and 2b. The spectrum of the calendula/okra (CO) extract (Fig. 1b) displayed characteristic bands for polysaccharides and phytochemicals: a broad O—H stretch at 3397 cm^−1^, C—H stretching at 2932 cm^−1^, carbonyl (C=O) stretching at 1725 cm^−1^, conjugated C=C at 1626 cm^−1^, carboxylate (COO^−^) stretching at 1445 cm^−1^, and C—O stretching at 1056 cm^−1^. The successful green synthesis of Ag NPs was indicated by the persistence of these bands, confirming the organic capping layer. The spectrum of Ag@HKUST-1 retained the key features of both precursors, with the appearance of new, distinct bands at 480 cm^−1^, 589 cm^−1^, and 720 cm^−1^, corresponding to the Cu—O vibrational modes of the HKUST-1 framework (Nivetha et al., 2020; Olfati et al., 2021; Konkal et al., 2026). This confirms the successful formation of the composite without the degradation of the organic components. UV–Vis spectroscopy provided further evidence for the synthesis of Ag NPs and the Ag@HKUST-1 composite (Fig. 4d). The biosynthesized Ag NPs exhibited a characteristic surface plasmon resonance (SPR) band at 440 nm, confirming the formation of nano-sized silver particles (Li et al., 2019). The HKUST-1 spectrum showed absorption below 460 nm due to π–π* transitions in its organic ligands (Di Credico et al., 2018; Tian et al., 2019). The spectrum of the Ag@HKUST-1 composite displayed a broad absorption band spanning 350–800 nm, a clear signature of the combined electronic transitions from the HKUST-1 framework and the SPR of the embedded Ag NPs (Li et al., 2019). The FTIR analysis of the polysaccharide derivatives (Fig. 2b) confirmed progressive functionalization. The spectrum of native OP showed characteristic peaks for O–H/N–H stretching (3433 cm^−1^), C—H stretching (2925 cm^−1^), amine groups (1639 cm^−1^), and C—O stretching (1091 cm^−1^) (de Jesus et al., 2013; Deka et al., 2023; Eydi Gabrabad et al., 2024). The successful introduction of aldehyde groups in DACMOP was evidenced by a new, sharp C=O stretching peak at 1735 cm^−1^ (Ghanbari et al., 2021). Critically, the spectrum of the final Hydrogel/Ag@HKUST-1 composite (Fig. 2b) revealed two key features: (i) a peak at 1629 cm^−1^, indicative of a Schiff base bond formed between the amine groups of the hydrogel network and the aldehyde groups of DACMOP, confirming effective cross-linking; and (ii) a broad band at 3439 cm^−1^, signifying strong hydrogen bonding (O–H/N–H), which contributes to the structural stability and swelling properties of the hydrogel (Nezhad-Mokhtari et al., 2024).

XRD patterns (Figs. 1c & 2c, 2θ = 5–75°) elucidated the crystalline nature of the components. The CO extract exhibited a broad, amorphous peak (15–35°), while the biogenic Ag NPs showed distinct crystalline peaks at 32.3°, 46.3°, 55.1°, 67.7°, and 76.8°, corresponding to the (111), (200), (142), (220), and (311) planes of face-centered cubic silver, respectively [39, 45, 51]. The synthesized HKUST-1 displayed sharp, characteristic diffraction peaks, most notably a high-intensity peak at 11.76°, confirming its high crystallinity (Nivetha et al., 2020). The XRD pattern of OP indicated a semi-crystalline structure with peaks around 2θ = 32°, 44°, and 56°. Its derivatives (CMOP, DACMOP, DCDACMOP) showed increased crystallinity and sharper peaks due to the introduction of aldehyde and carboxylate functional groups. The pure hydrogel matrix was amorphous, exhibiting a broad diffraction band between 25° and 45°. Importantly, the XRD pattern of the final Hydrogel/Ag@HKUST-1 composite retained this amorphous background but also displayed weak crystalline peaks corresponding to the embedded Ag@HKUST-1 nanoparticles. This confirms the successful integration of the crystalline nanocomposite into the amorphous hydrogel network without altering its primary phase, thereby enhancing the overall structural complexity.

### 3.2 Morphology studies

The surface morphology and composition of the synthesized materials were examined using SEM, EDX, and elemental mapping, as illustrated in Fig. 1a and Fig. 2a. In Fig. 1a, the SEM analysis of the CO extract revealed a smooth, uniform surface without significant nanostructures, indicating the amorphous nature. The SEM image of green-synthesized and stabilized Ag NPs with CO extract shows Ag NPs with 10–25 nm particle size with enhanced surface roughness, complexity. The HKUST-1 SEM image exhibits a crystalline structure with a polyhedral morphology (Al-Janabi et al., 2015). The Ag@HKUST-1 SEM image indicates that incorporating Ag NPs into the HKUST-1 matrix altered polyhedral morphology to a uniform morphology, and the EDX spectrum of Ag@HKUST-1confirms the presence of copper (Cu) from HKUST-1 along with Ag particles. In Fig. 2a, SEM images of the hydrogel revealed an amorphous structure with an interconnected network. However, combining Ag@HKUST-1 with a hydrogel matrix substitutes its amorphous structure with a semi-crystalline structure. EDX/MAP further confirms the successful preparation of Hydrogel/Ag@HKUST-1. The gradual degradation of the semi-crystalline structure of Hydrogel/Ag@HKUST-1 during wound healing facilitates the continuous release of Ag^+^ and Cu^2+^ ions, which are crucial for bacterial eradication through the disruption of microbial membranes (Lu et al., 2023; Urnukhsaikhan et al., 2021). The SEM findings underscore the structural advantages of integrating Ag NPs and HKUST-1, resulting in a 3D structure ideal for prolonged wound care and therapeutic efficacy.

### 3.3 In vitro swelling and degradation behavior

**Fig. 3:**
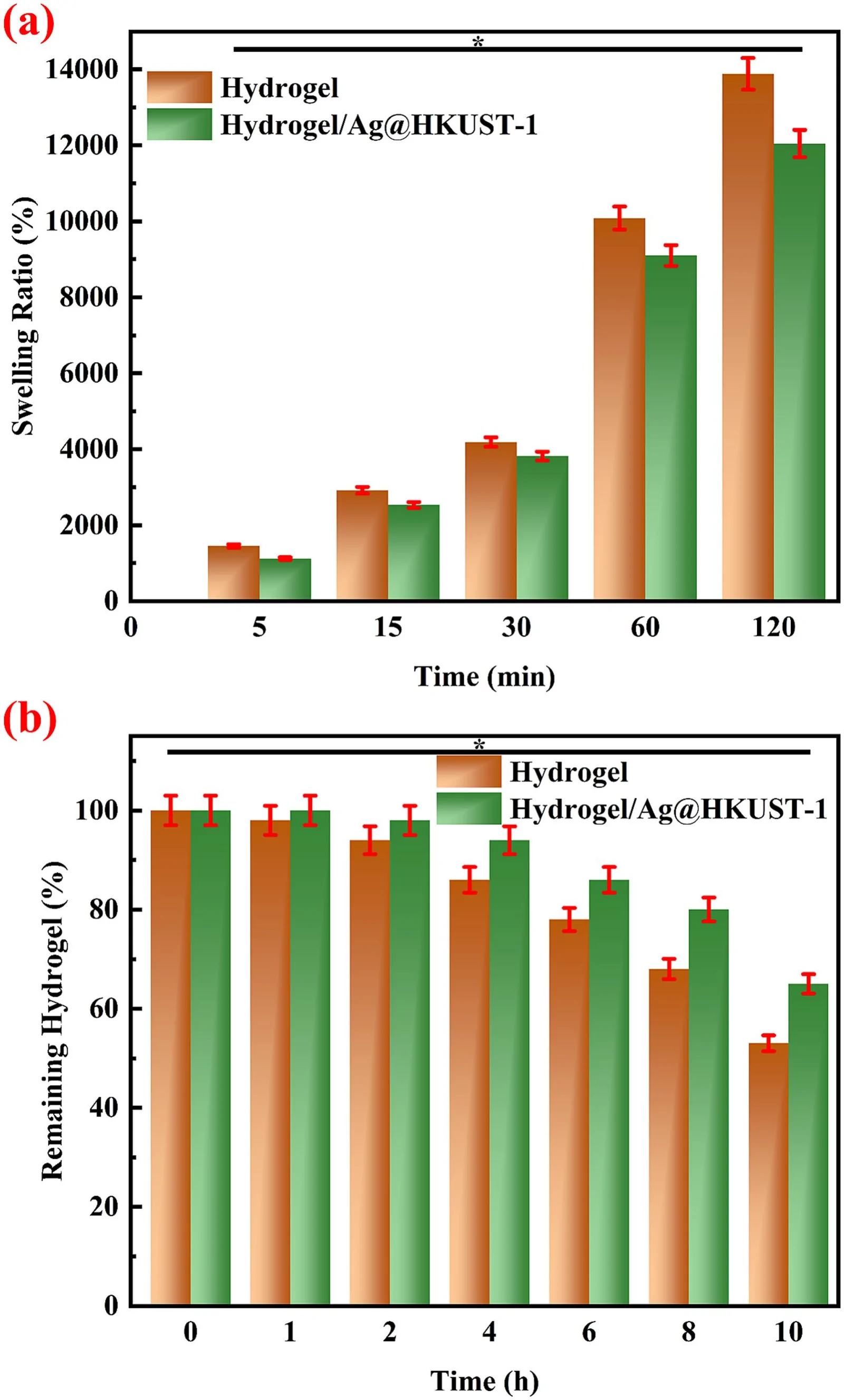
a) Swelling percentage of the synthesized hydrogel and Hydrogel/Ag@HKUST-1 at pH 7.4. b) In vitro degradation behavior of the hydrogel and Hydrogel/Ag@HKUST-1 (*n* = 3, *p < 0.05).

Fig. 3a illustrates the swelling behavior of the hydrogel and Hydrogel/Ag@HKUST-1 in PBS at 37 °C. Efficient swelling allows dressings to swiftly absorb wound exudate, which is vital for keeping a moist environment around the wound, reducing the risk of bacterial infection, promoting cell proliferation, and facilitating nutrient exchange (Feng and Wang, 2023; Wang et al., 2021). Furthermore, in a wound affected by bacterial contamination, the elevation of pH levels causes the hydrogel to swell, which enlarges its pore size and promotes the release of the antimicrobial agent (Ag^+^ and Cu^2+^ ions) into the surrounding medium, thus aiding in the management of the infection (Ribeiro et al., 2024). Fig. 3a shows that all hydrogel formulations demonstrated a rapid swelling response during the initial 5 min. This high swelling ratio may result from a lower crosslinking density, along with the modification of OP, which incorporates hydrophilic groups into polymer chains and enhances superior liquid absorption capacity (Hoop et al., 2018; Kaya and Derman, 2023). After 2 h, the hydrogel swelling ratio is 13.88 × 10^3^%. In the case of hydrogel/Ag@HKUST-1, the swelling ratio is 12.04 × 10^3^%, which the reduction in swelling ratio is due to the interaction between Ag@HKUST-1 and the hydrogel network. This observation underscores the significant influence of crosslinking density on the swelling behavior of hydrogel networks (Hoop et al., 2018).

In biomedical applications, accurately managing the degradation rate of scaffolds is essential. Consequently, Fig. 3b illustrates the in vitro degradation behavior of hydrogel and Hydrogel/Ag@HKUST-1. The hydrogel displayed the fastest degradation rate because of its reduced crosslinking density. Nevertheless, the degradation rate of Hydrogel/Ag@HKUST-1 has decreased, as Ag@HKUST-1 is physically crosslinked with the hydrogel network, enhancing structural stability and integrity (Zhang et al., 2014).

### 3.4 Adhesive properties

**Fig. 4:**
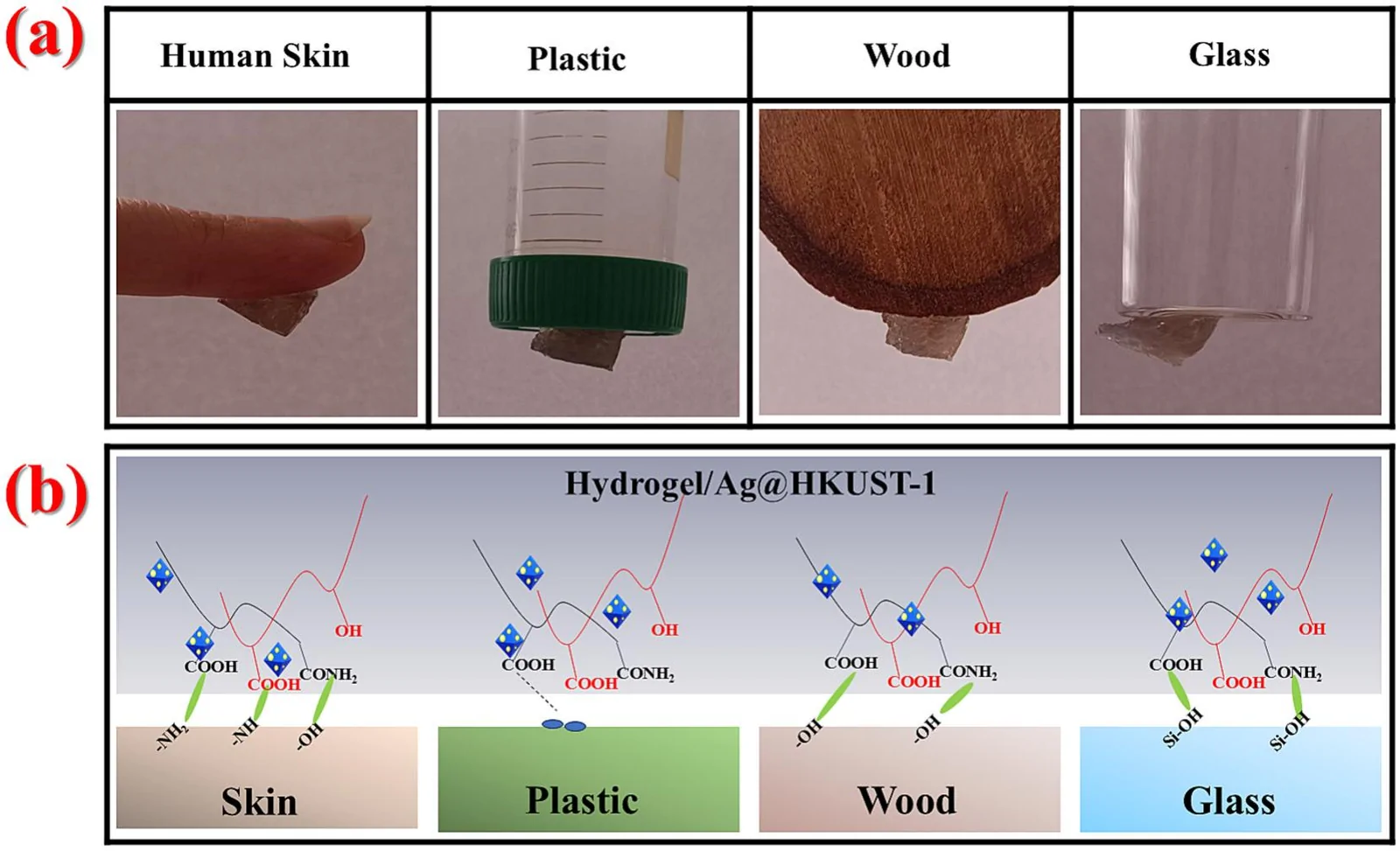
a) Adhesion properties of Hydrogel/Ag@HKUST-1on human skin and various surfaces. b) The adhesion mechanism for Hydrogel/Ag@HKUST-1.

The adhesive properties of wound dressings are essential for swift wound closure, hemostasis, and accelerating recovery (Rong et al., 2025; Zhang et al., 2020). Fig. 4 demonstrates the adhesive properties of Hydrogel/Ag@HKUST-1 on various surfaces, including human skin, wood, glass, and plastic. The findings exhibited enhanced adhesion properties across all evaluated surfaces due to the hydrogel matrix's hydrogen bonding interactions and the substances' surface. Mechanisms like aryl-aryl coupling and Michael addition significantly improve the adhesion strength by promoting robust interactions between hydroxyl (-OH) and amino (-NH₂) groups of Hydrogel/Ag@HKUST-1 and surfaces with amine or thiol groups along with electrostatic and hydrogen bonding interactions of carboxyl (-COOH) group with various substrates (El-Sayed et al., 2024). The durable adhesion, which is beneficial for biomedical applications, is enhanced by the stable amide linkages formed between the aldehyde groups of Hydrogel/Ag@HKUST-1 and the amino groups of tissue (Dong et al., 2024; Gao et al., 2019; Pelin et al., 2023; Wan et al., 2022).

### 3.5 Antibacterial

**Fig. 5:**
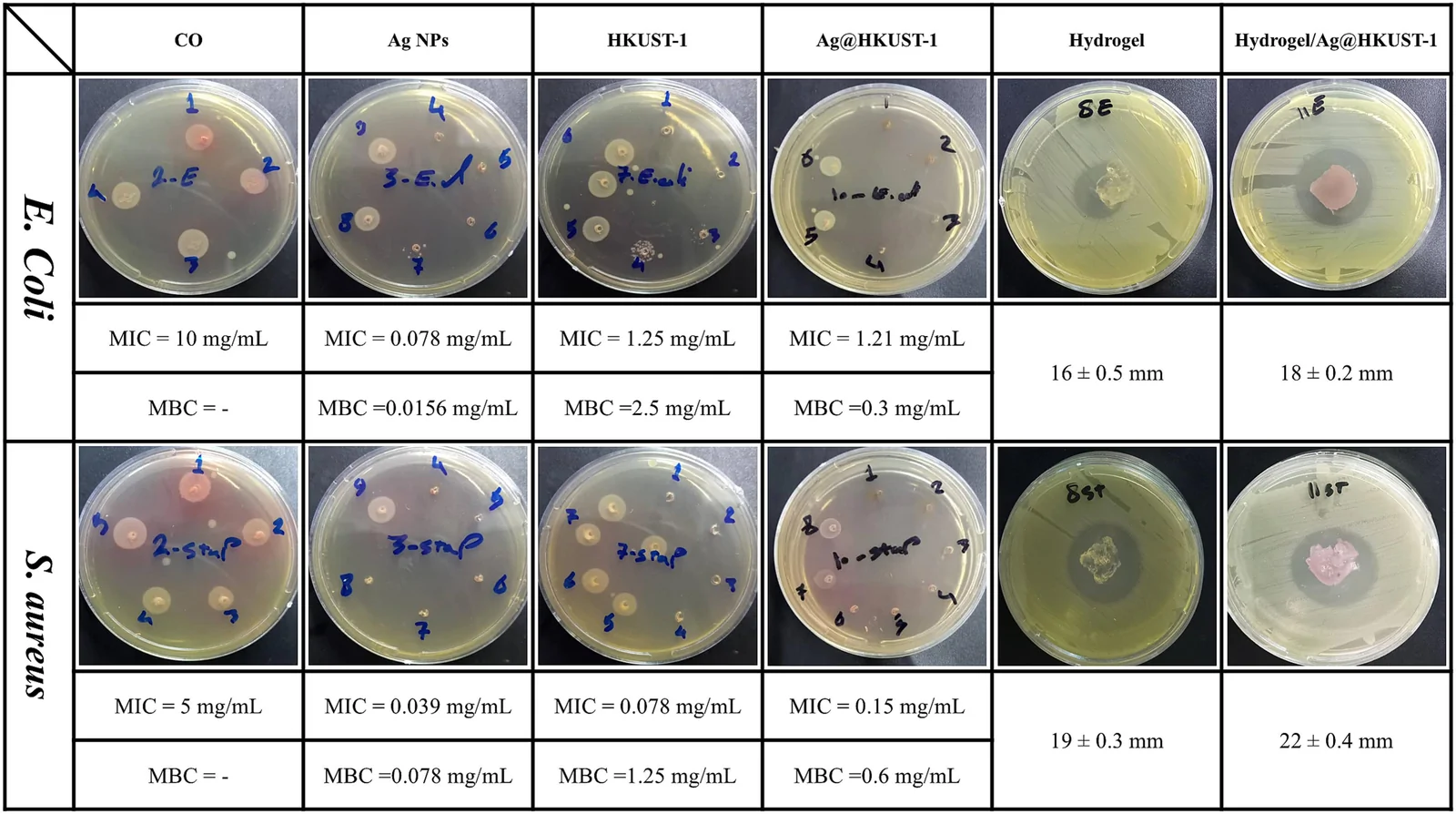
The antibacterial activity of CO extract, Ag NPs, HKUST-1, and Ag@HKUST-1 hydrogel and Hydrogel/Ag@HKUST-1 against *E. coli* and *S. aureus*.

The antimicrobial potential of the CO extract, Ag NPs, HKUST-1, and the Ag@HKUST-1 composite was quantitatively assessed using minimum inhibitory concentration (MIC) and minimum bactericidal concentration (MBC) assays. Subsequently, the antibacterial performance of the final hydrogel and Hydrogel/Ag@HKUST-1 composite was qualitatively evaluated against *S. aureus* (Gram-positive) and *E. coli* (Gram-negative) via the agar well diffusion method (Fig. 5). Ag NPs demonstrated the strongest activity, with notably low MIC values of 0.078 mg/mL against *E. coli* and 0.039 mg/mL against *S. aureus*. The composite Ag@HKUST-1 also showed significant efficacy. In the diffusion assay, the Hydrogel/Ag@HKUST-1 composite formed larger zones of inhibition than the pure hydrogel against both pathogens: 22 ± 0.4 mm versus 19 ± 0.3 mm for *S. aureus*, and 18 ± 0.2 mm versus 16 ± 0.5 mm for *E. coli*. This enhanced antibacterial action is attributed to a synergistic multi-mechanistic effect. The functional aldehyde groups within the composite disrupt bacterial cell integrity, leading to lysis. This effect is augmented by the hydrogel's Schiff base and carboxyl groups (Ceramella et al., 2022; Ul Haq et al., 2024; Zi et al., 2018). Additionally, Cu^2+^ ions released from the MOF framework interact with bacterial membranes, causing destabilization and dysfunction (Edlo and Akhbari, 2023; Lv et al., 2025). Moreover, Ag NPs release Ag^+^ ions that interact with bacteria membrane proteins negatively charged thiol groups, leading to membrane destabilization and apoptosis in bacteria (Paul et al., 2025; Zhu et al., 2024). The enhanced antibacterial activity of Hydrogel/Ag@HKUST-1 can be attributed to the synergistic effects of Ag^+^ and Cu^2+^ release, along with the antimicrobial contribution of aldehyde groups and Schiff base linkages. These combined interactions improve the antibacterial performance of the hydrogel, highlighting its potential as an effective multifunctional wound dressing material.

### 3.6 Antioxidant activity

**Fig. 6:**
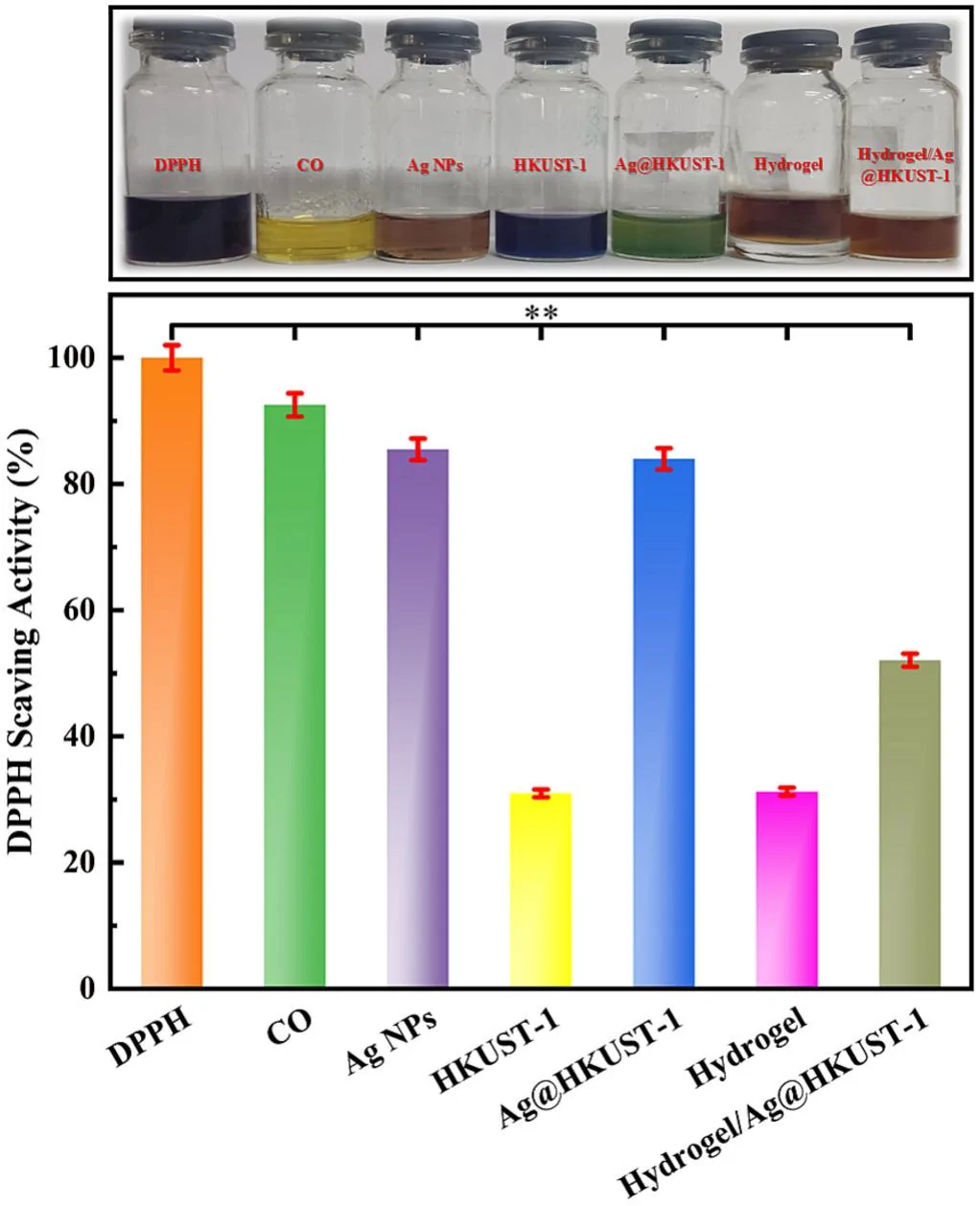
Percentage of scavenging effect of prepared samples along with their antioxidant activity image. (*n* = 3, **p < 0.01).

Effective antioxidant strategies are critical during wound repair. The heightened inflammatory response releases excess cytokines, which escalate the generation of reactive oxygen species (ROS) and elevate oxidative stress. This condition can disrupt the natural healing cascade and exacerbate tissue injury (Hennia et al., 2018). The antioxidant performance of the materials, as assessed via the DPPH radical scavenging assay, is shown in Fig. 6. The CO extract demonstrated exceptional efficacy, neutralizing 92.54% of DPPH radicals due to its rich composition of flavonoids, carotenoids, and triterpenoids, which collectively mitigate oxidative stress and inflammation by scavenging ROS (Ejiohuo et al., 2024). The tested nanomaterials and hydrogels also exhibited significant free radical scavenging activity, with Ag NPs, Ag@HKUST-1, and the final Hydrogel/Ag@HKUST-1 composite achieving efficiencies of 85.5%, 84.00%, and 52.06%, respectively, highlighting their therapeutic potential for managing oxidative stress in wound environment. The antioxidant activity observed in this study agrees with recent reports where antioxidant molecules combined with Ag nanoparticles improved wound microenvironment regulation (Nabilah et al., 2025).

### 3.7 Hemocompatibility and blood clotting evaluations

Since the interaction between biomaterials and red blood cells (RBCs) is essential, hemolysis assay was used to examine hydrogel and Hydrogel/Ag@HKUST-1. Materials are classified by their hemolysis rate into three groups: hemolytic (over 5%), slightly hemolytic (2 to 5%), and non-hemolytic (below 2%) (Weber et al., 2018). In this study, RBCs were subjected to varying concentrations of Ag@HKUST-1 (5, 10, and 15%) within a hydrogel matrix for 1 h at 37 °C. With Ag@HKUST-1 at its highest concentration of 15%, the hemolysis rate remained below 0.72%, lower than the usual values for hemostatic materials. The hydrogel exhibited the lowest hemolysis rate, releasing just 0.15% hemoglobin. This finding highlight hydrogel and Hydrogel/Ag@HKUST-1`s excellent biocompatibility and non-toxicity to red blood cells.

**Fig. 7:**
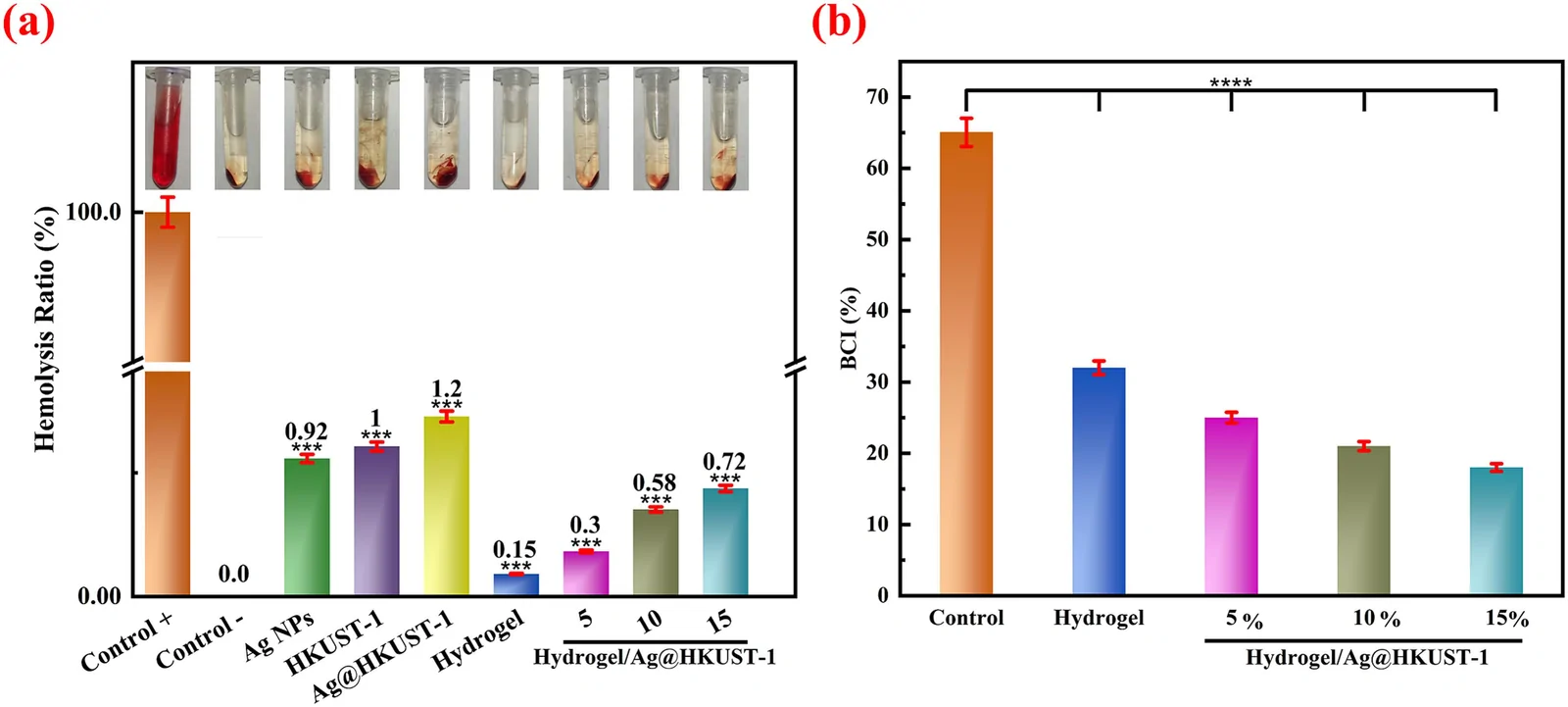
a) Hemolytic activity assessment to examine the biocompatibility of the samples. b) The whole blood clotting index analysis for the hydrogel and Hydrogel/Ag@HKUST-1. (*n* = 3; ***p < 0.01, ****p < 0.05).

Advanced wound dressings demand enhancing blood clotting. Blood coagulation constitutes a multifaceted biological mechanism that necessitates the synchronized functions of coagulation factors, fibrinogen, platelets, and the vascular endothelium, alongside their interactions with the fibrinolytic system (Nezhad-Mokhtari et al., 2024; Zhang et al., 2023). Fig. 7b demonstrates the blood clotting index (BCI) of hydrogel and Hydrogel/Ag@HKUST-1 (with 5, 10, and 15% concentrations of Ag@HKUST-1) through a dynamic whole-blood clotting test model. Hydrogel/Ag@HKUST-1 demonstrated a notable improvement in blood clotting performance relative to the hydrogel, exhibiting a significant decrease in clotting time with increasing Ag@HKUST-1 concentration. In hydrogel and Hydrogel/Ag@HKUST-1, PAA's negatively charged carboxyl groups activate blood coagulation factor XII and PAA's hydrophilic properties enhance water absorption, which promotes rapid and stable clot formation within the hydrogel network (Tan et al., 2023; Wan et al., 2022). The reduced blood clotting time in Hydrogel/Ag@HKUST-1 is attributed to Ag NPs, which result from the phytochemicals in CO extract acting as capping agents on the surface of the biosynthesized Ag NPs, along with the negative charges associated with these NPs (Abdoli et al., 2024; Ayyanahalli Matta et al., 2022). These findings suggest that Hydrogel/Ag@HKUST-1 could serve as a promising alternative to conventional wound care materials such as gauze. Likewise, recent studies on Ag NP-based multifunctional wound dressings have also demonstrated their ability to simultaneously provide antibacterial activity, hemostatic performance, and tissue regeneration, further supporting the potential of multifunctional platforms for advanced wound management (Wang et al., 2025).

### 3.8 Cytotoxicity evaluation

**Fig. 8:**
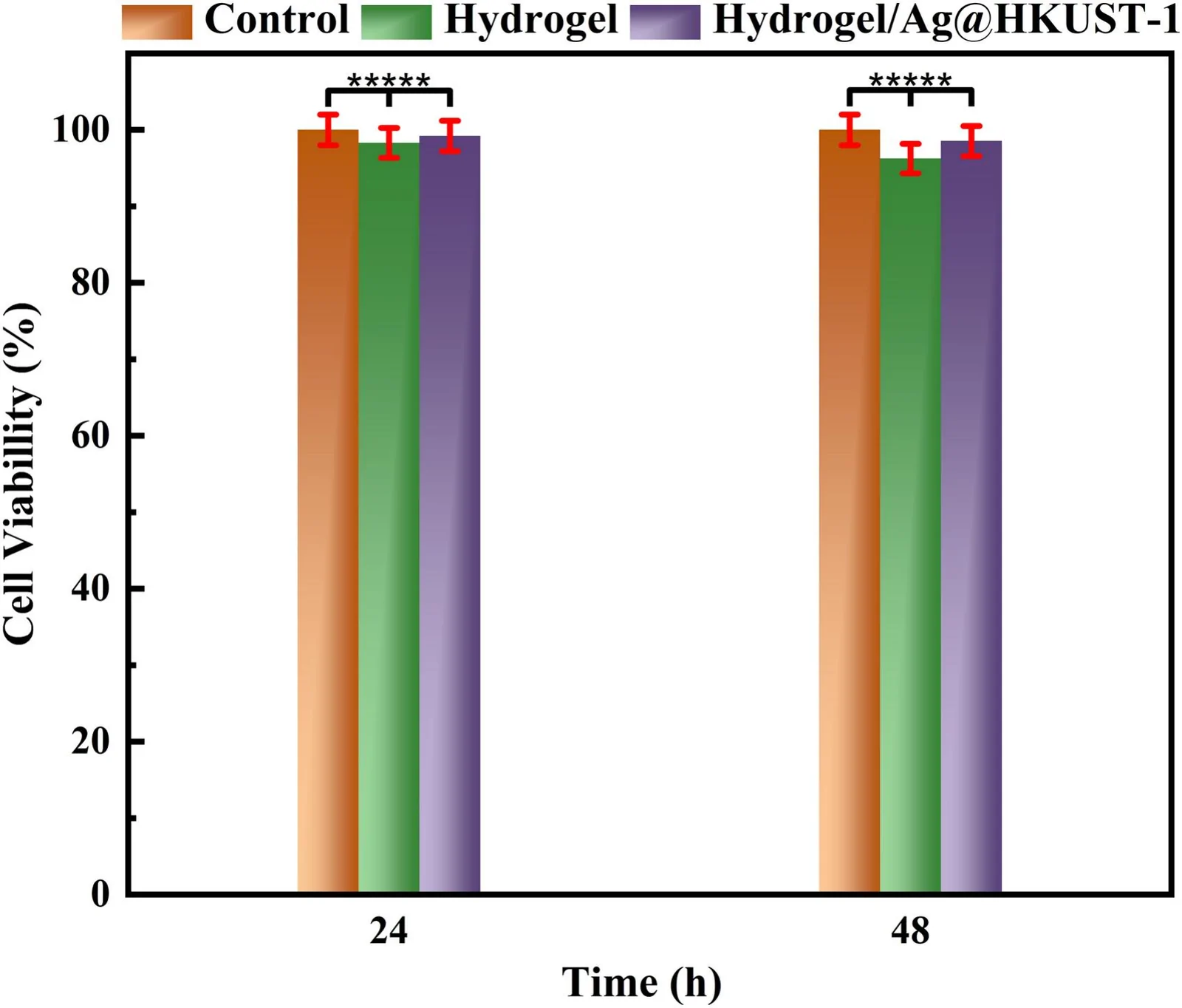
Cell viability of HFF-2 after exposure to hydrogel and Hydrogel/Ag@HKUST-1. (*n* = 3, *****p < 0.05).

The cytocompatibility of the prepared hydrogels was evaluated using an MTT assay on HFF-2 cells under the intended application conditions (Fig. 8). Both hydrogel and Hydrogel/Ag@HKUST-1 exhibited high cell viability, with values above 97% after 48 h, confirming their excellent biocompatibility. The incorporation of HKUST-1 and green-synthesized Ag NPs stabilized by CO extract did not negatively affect HFF-2 cell survival and may contribute to the enhanced biological performance of the hydrogel. This favorable response can be attributed to the synergistic effects of Cu^2+^ ions, which promote fibroblast and keratinocyte activity through growth factor regulation, Ag NPs that support controlled bioactive release, and CO extract that facilitates fibroblast proliferation and migration. These findings are consistent with previous reports on Ag nanoparticle-containing natural polymer hydrogels, highlighting Hydrogel/Ag@HKUST-1 as a safe and promising candidate for wound healing applications (Li et al., 2025).

## 4 Conclusion

This study successfully developed a Hydrogel/Ag@HKUST-1 composite hydrogel with promising attributes for advanced wound dressing applications. Structural characterization confirmed the effective incorporation and homogeneous distribution of bioactive components, enhancing the hydrogel's functional integrity and stability. The hydrogel exhibited favorable swelling behavior, ensuring adequate exudate absorption and sustained moisture retention, both critical factors for promoting tissue regeneration. Enhanced antibacterial and antioxidant properties were achieved by incorporating bioactive HKUST-1 and green-synthesized Ag NPs using CO extract, which aids in preventing infections and minimizing oxidative stress in the wound environment. Hemocompatibility assessments confirmed excellent blood compatibility, with no detectable hemolytic effects. Furthermore, the hydrogel demonstrated pro-coagulant activity conducive to accelerated wound closure, while MTT assays revealed minimal cytotoxicity and high biocompatibility. Overall, Hydrogel/Ag@HKUST-1 represents a promising multifunctional and biocompatible platform for advanced wound dressing applications.

## 5 Limitations and future perspectives

Although Hydrogel/Ag@HKUST-1 exhibited promising physicochemical, antioxidant, antibacterial, and cytocompatible properties based on in vitro evaluations, further studies are required to confirm its therapeutic potential. Future investigations will include in vivo wound healing models to assess tissue regeneration, angiogenesis, collagen deposition, inflammatory responses, and histological changes. Moreover, detailed analyses of concentration-dependent cytotoxicity, intracellular ROS regulation, oxidative stress biomarkers, enzymatic degradation, and the individual contribution of Ag and HKUST-1 components through ion-release profiling, membrane permeability assays, and selective inhibition studies will provide deeper insights into the biological performance and underlying mechanisms of this system.

## CRediT authorship contribution statement

**Shabnam Tahmasebi:** Writing – original draft, Visualization, Methodology, Investigation, Formal analysis, Data curation, Conceptualization. **Hassan Farmanbordar:** Writing – original draft, Visualization, Software, Project administration, Methodology, Investigation, Formal analysis, Data curation, Conceptualization. **Reza Mohammadi:** Writing – review & editing, Validation, Supervision, Resources.

## Declaration of competing interest

The authors declare that they have no known competing financial interests or personal relationships that could have appeared to influence the work reported in this paper.

## Data Availability

Data will be made available on request.
