# Supplementary material for: Antibacterial and antioxidant okra-based hydrogels enhanced with Ag@HKUST-1 as a next-generation wound dressing
Source: Int J Pharm X. 2026 Aug 20;12:100633. doi: 10.1016/j.ijpx.2026.100633 (PMC13551895; doi:10.1016/j.ijpx.2026.100633)
Supplement: Supplementary file 1 [file mmc1.docx]

**Supporting Information**

**Antibacterial and Antioxidant Okra-Based Hydrogels Enhanced with Ag@HKUST-1 as a Next-Generation Wound Dressing**

Shabnam Tahmasebi, Hassan Farmanbordar, Reza Mohammadi*

*Polymer Research Laboratory, Department of Organic and Biochemistry, Faculty of Chemistry, University of Tabriz, Tabriz, Iran*

* Corresponding author

E-mail address: r.mohammadi@tabrizu.ac.ir (Reza Mohammadi)

**Supplementary Materials and Methods:**

**1. Characterization**

Samples' chemical structure was assessed with a Bruker Tensor 27 (Germany) FT-IR spectroscopy over a 400–4000 cm⁻¹ range. Bruker D8 Advanced diffractometer was used to investigate the synthesized materials ' phase purity and compound patterns at ambient temperature. Shimadzu UV-2100 (Japan) spectrophotometer analyzed the synthesized nanoparticles (NPs) optical properties (at ambient temperature and wavelength = 200 to 800 nm. LEO 1455VP SEM was utilized to investigate Samples`s structural and morphological characteristics by applying a thin layer of gold (~10 nm) to enhanced conductivity with working distance from 2.71 to 3.58 cm at an accelerating voltage of 15 kV. ImageJ software analyzed particle size distribution on multiple SEM images. The samples' elemental composition and distribution were measured using energy-dispersive X-ray spectroscopy (TESCAN VEGA (Czech Republic)). Buffer solution pH values under standard conditions were assessed using an INESA calibrated PHS-3C pH meter (China).

**2. Extraction of OP**

For OP extraction the peel was separated from the seeds. The dried peel was chopped and at 50 ºC soaked in a 5 % NaCl solution overnight. Then the fabric filter was utilized to separate the mucilage. The separated mucilage was washed with 9:1 ethanol/ isopropanol solvent at ambient temperature to extract OP and dried in an oven at 50 ºC.

**3. Determining the carboxymethylation content of CMOP**

Conductometric titration was utilize to determine carboxymethylation of CMOP to assess carboxyl group modification (Lin et al., 2021; Stojanović et al., 2005). To prepare a 0.05 % (w/v) OP solution, a defined quantity of CMOP was dissolved in 50 mL of distilled water. To protonate the carboxylic acid groups concentrated HCl was added. The solution was titrated with 0.1 M NaOH in presence of methyl orange indicator. The carboxymethylation degree obtained to be approximately 78 %. Triplicate analyses (n = 3) were conducted, and the carboxymethylation degree was calculated using Eq. (1):

$Degree Carbomethylation=\frac{\text{ }\text{V}_{\mathrm{NaOH}}\text{×}C_{\mathrm{NaOH}}}{M_{\mathrm{CMOP}}}$	(1)

V _NaOH_, C _NaOH_, and M _CMOP_ are NaOH consumed volume (mL), NaOH concentration (mol/L), and the dry weight of sample (g), respectively.

**4. Determining the dialdehyde content of DACMOP**

Dialdehydes' formation was quantified using a modified titration technique involving hydroxylamine hydrochloride (NH_2_OH.HCl) as depicted in Figure 1 (Lucas de Lima et al., 2020; Sichinga et al., 2022). 0.1 g of DACMOP was solubilized in 25 mL of a 0.25 M NH_2_OH·HCl solution and subsequently adjusted to a pH of 3.5 utilizing 1 M HCl. The resultant reaction mixture was subjected to stirring at ambient temperature overnight to facilitate the conversion of aldehyde functional groups to oximes (C=N–OH). The released HCl was titrated using 0.1 M NaOH, alterations in pH were meticulously documented throughout the titration process. The oxidation efficiency was approximately 80 %. Triplicate analyses (n = 3) were performed, and the oxidation efficiency was calculated using Eq. (2):

$$Degree \mathrm{Dialdehyde}=\frac{C_{NaOH}V_{NaOH}}{12.5 m}\times100 (2)$$

C*_NaOH_*, V*_NaOH_*, and *m* are the concentration of NaOH (mol/L), NaOH consumed volume (mL), and the DASA sample mass (g), respectively.

**5. Determining the decarboxylation content of DCDACMOP**

Titration was used to evaluated the decarboxylation degree in DCDACMOP (Lin et al., 2021; Stojanović et al., 2005). A 0.05 g solution of DCDACMOP was prepared by dissolving it in 50 mL of distilled water. To protonate the carboxylic acid groups concentrated HCl was added. The solution was titrated with 0.1 M NaOH in presence of methyl orange indicator. The carboxymethylation degree obtained to be approximately 79 %. Triplicate analyses (n = 3) were conducted, and the carboxymethylation degree was calculated using Eq. (3):

$Degree \mathrm{Dicarboxylation}=\frac{\text{ }\text{V}_{\mathrm{NaOH}}\text{×}C_{\mathrm{NaOH}}}{M_{DCDACMOP}}$	(3)

V_NaOH_, C _NaOH_ and M _DCDACMOP_ are NaOH consumed volume (mL), NaOH concentration (mol/L), and the dry weight of sample (g), respectively.

**6. Extraction of COE**

To remove impurities, 5 g of CO was washed with distilled water and dried at an ambient temperature. In 100 mL of a 1:2 ethanol-distilled water mixture, the dried material was immersed at 60 °C and stirred overnight. Residual solids were removed using filter paper, centrifuged to purify the CO extract, freeze-dried, and stored at -4 °C. The extracted CO was used for the green synthesis of Ag NPs.

**7. Synthesis of HKUST-1 MOF**

1.26 g (7.07 mmol) of benzene-1,3,5-tricarboxylic acid and 3.52 g (12.66 mmol) of copper (II) nitrate hemi pentahydrate were dissolved in a 100 mL solvent mixture made of equal parts N, N-dimethylformamide (DMF), ethanol, and deionized water. The reactants were stirred for 15 min at room temperature before being placed in an oven at 85 ºC for 20 h. The product was washed with DMF, resulting in blue HKUST-1 MOF crystals (Nivetha et al., 2020).

**References**

Lin, C., Chen, B., Liu, Y., Chen, Y., Liu, M., Zhu, J., 2021. Carboxylated cellulose nanocrystals with chiral nematic property from cotton by dicarboxylic acid hydrolysis. Carbohydrate Polymers 264, 118039.

Lucas de Lima, E., Fittipaldi Vasconcelos, N., da Silva Maciel, J., Karine Andrade, F., Silveira Vieira, R., Andrade Feitosa, J.P., 2020. Injectable hydrogel based on dialdehyde galactomannan and N-succinyl chitosan: a suitable platform for cell culture. Journal of Materials Science: Materials in Medicine 31, 1–13.

Nivetha, R., Sajeev, A., Paul, A.M., Gothandapani, K., Gnanasekar, S., Bhardwaj, P., Jacob, G., Sellappan, R., Raghavan, V., Pitchaimuthu, S., 2020. Cu based Metal Organic Framework (Cu-MOF) for electrocatalytic hydrogen evolution reaction. Materials Research Express 7, 114001.

Sichinga, M.C., Kline, T., Whitehead, M., van de Ven, T.G., 2022. One-pot eco-friendly oxidative synthesis of imine carboxymethyl dialdehyde cellulosic fibers. Cellulose, 1–17.

Stojanović, Ž., Jeremić, K., Jovanović, S., Lechner, M.D., 2005. A comparison of some methods for the determination of the degree of substitution of carboxymethyl starch. Starch‐Stärke 57, 79–83.
